# Books Reviewed

**Published:** 1868-10

**Authors:** 


### Editorial Department.

### Books Reviewed.

Diseases Peculiar to Women, including Displacement of the Uterus. By Hugh L. Hodge, M. D. Henry C. Lea: Philadelphia, 1868.

The first edition of this work was prepared mainly for the Alumni of the University of Pennsylvania; the object, as stated by the author, being to "present, more at length, and in detail, those views on the nervous diseases of women, which he had for many years taught in the halls of the University." He says: "As the subject of metritis, in its various modifications and complications, has given rise to numerous discrepancies, both in theory and practice, the chapter on uterine inflammation has been greatly altered and enlarged. The object of the author was not merely to present what he deems a more correct theory and practice in inflammatory diseases of the uterus, but also to insist that a very large proportion of the so-called cases of metritis are, in reality, but examples of irritation, where inflammation has subsided, or where it has actually never existed. Indeed, the chief object of this whole work is to exemplify the nature, consequences, and treatment of nervous irritation, as distinct from inflammation. In carrying out this idea, the author has endeavored to show that the nervous diseases of women, often grouped under the word "hysteria," can be generally traced to the pelvic viscera, and especially to the uterus. The causes of such disturbances, whether centric or eccentric, are exceedingly diversified—the nervous susceptibility of women being easily disturbed by innumerable moral and physical excitements, experience has convinced the author that not only is the uterus involved in most of these complaints, but that its disturbances are very frequently dependant upon displacements of the organ, however induced. Hence, special attention has been given to this subject of displacements of the uterus; the more so, as, in the opinion of the author, these displacements of the uterus are of great importance, and have, as he must think, been seldom treated on scientific principles."

We have quoted, quite at length, the authors preface, thus hoping to give our readers an idea of the character and design of the work. And we will now turn over and learn, if possible, what principles of treatment in uterine displacements are scientific. We quite agree with the author, that they have seldom been treated upon such principles, and we have yet to learn what efficient and rational means may be resorted to for relief.

The first indication is, "to remove or palliate any existing causes." The second, "to replace the uterus in its natural position." The third, "to *retain* it when thus replaced." In substance, he says: In almost all cases of *procidentia* the uterus can be readily reduced, so as, at least, to enter the pelvis. In cases of retroversion, and especially of retroflection, it is more difficult. In some instances this can be accomplished by the finger, alone, in the vagina or rectum. This is not practicable in women who have not borne children. Elevators, the gumelastic bag, and other instruments, acting per rectum or per vaginum, may sometimes succeed. The uterine sound or probe of suitable size and curve, and a suitable shaped pessary, the only efficient instruments for restoring the uterus to its normal position. In very few cases, however, will any advantage result from the mere replacement of the organ when no measures are adopted to keep it *in situ*. To maintain the uterus in its normal position, is a problem of great difficulty, yet, perhaps, to be completely solved; for, after all the attention, ingenuity and science, which have been directed to this point, no suggestion has received the general sanction of the profession. Rest has been carried on for months and years, not only without relief, but with aggravation of all the symptoms. Rest, conjoined with astringents, is entirely insufficient; astringent washes are altogether uninfluential upon the position of the uterus in the pelvis. The theory that contraction of the vagina is requisite to the support of the uterus has been carried to a serious and dangerous practice. Diéffenbach, Gérardin, Bérard, and many others, in Europe and America, have resorted to the knife to fulfill this false indication. External bandages have no tendency to restore the organ to its normal position. Internal pessaries receive from our author full consideration, and all the various machines used for that purpose are illustrated, and the manner of introduction described. Pessaries are an especial horror to us, for we have rarely known much good of them, and almost always, harm has resulted from their use. Dr. Hodge speaks of them with some favor.

We have followed the author along through this subject, hoping to find something worth knowing, but alas! the means for preserving a displaced uterus in its natural position is yet to be discovered. This should teach physicians the absurdity of day after day replacing it with probes, sounds, etc. Would it not generally be better to remember that displacement may exist with impunity, and let it remain where it is found until we have means of retaining it in position when replaced.

The work is written in clear, direct and beautiful style; and the author maintains his opinions with the most lucid and convincing arguments. There is room for differences of opinion, but the positions are all well taken, and sustained with sufficient proofs. We most heartily commend this work to the careful perusal of all interested in the subject, believing that the general soundness of the author's views will be thus fully apparent.

---

A Theoretical and Practical Treatise on Midwifery, including the Diseases of Pregnancy. By P. Cazeaux. Lindsay and Blakiston: Philadelphia, 1868.

We are happy to announce the appearance of the Fifth American, from the Seventh French, Edition of Cazeaux's Widwifery, a work which is complete in all respects, and which has been received with universal favor, by the profession, in all countries. Every topic within the range of its design has been considered. and the accuracy and soundnes of its teachings are unsurpassed. There is no better text-book for the student, and no safer guide for the practitioner; it embodies the latest and best views in pathology, and the most safe and successful modes in practice. It is also illustrated with one hundred and seventy-five wood cuts, which add greatly to the value of the work and make it much better adapted to the wants of the medical student. It is quite unnecessary for us to go into any review of the text, as all our readers are too familiar with the character of the work. The simple announcement of a new edition is quite enough for most purposes, but yet, it may be well to say that the work has been subjected to revision by Prof. Tamier, since the death of Cazeaux, who gives the following explanations in his preface to the Seventh, and last, French Edition:

"A classical book soon grows old in these days, and it was found impossible to bring out a new edition without subjecting it to the alterations demanded by the progress of science. I was charged with its preparation, and accepted the honor of the task with a full appreciation of its difficulties. I was left at liberty to remodel the work according to my judgment; to make the alterations which seemed to be required; to suppress some passages, and to introduce new ones."

"Out of respect to Cazeaux's memory, it was decided that the printing should be done in two kinds of type: the larger for the old text, and the smaller for what I had written myself. * * * I have, therefore, reviewed and corrected all parts of it with scrupulous care. * * * Chapters entirely new will be found in it on the diseases of pregnancy, the alterations to which the placenta is subject, and the death of the child during intra-uterine life. * * * In the study of the accidents which are liable to complicate labor, I have profited by all the works published of late years, and in the account of hemorrhage, puerperal convulsions, and the indications which they present, will be found some new considerations."

We cannot quote more fully from the editor's preface, and have failed to give an adequate idea of the improvements, alterations and revisions the work has undergone. Suffice it to say, that as now offered, it is a work of unsurpassed excellence.

---

Aitkin's Science and Practice of Medicine. Second edition. Vol. 1. Philadelphia: Lindsay & Blakiston.

The present state of medical science is, perhaps, more fully presented in this work than in any other, and its reception by the American profession is clearly shown in the appearance of the second edition in so short a period after its first publication in this country.

The English authors have generally presented us the science of medicine in unexceptionable manner, but to follow their practice in this country has been quite inconsistent with our present therapeutical views. Watson describes disease very accurately and beautifully, but we cannot imitate his practice. There is still too much of the "heroic practice," as it was formerly called, in most foreign authors who have written upon the treatment of disease, and we require an American editor, before we can receive their works as adapted to either our views or wants.

Aitkin's science and practice of medicine is intended to cover the whole field, and to form a guide in the diagnosis and treatment of all maladies liable to fall under the care of the physician. The additions made in this edition were spoken of in our last number, and we have no occasion to repeat what we then said as to the completeness of the work in its present form.

The additions by Meredith Clymer, M. D., American editor, add very materially to the completeness and value of the work, and as now presented, it is truly a book of rare excellence, adapted both to the student and practitioner. When we have said all this in its favor we should feel quite at liberty to review its its ground, and if time and space would permit, would discuss briefly some parts of its teaching; this, however, for the present we must omit. The work is full of the opinions of others, and often the ideas of the author are only indicated by the quotations. In the treatment of typhoid fever he half recommends, 1st, Digitalis by quotations from Wunderlich. 2d, Calomel by Wunderlich, Wood, Parks and others. Stimulants, tonics, astringents, rubefacients, and all other remedies are presented and sustained by quotations. This is all very polite, but those who read Aitkin's practice would prefer to know what he thinks about it himself. The whole materia medica nearly has been thought useful in the treatment of typhoid fever by very good physicians, and even some have thought that counter-irritation was capable of breaking it up and curing it all together, but no one wants to know who believes in such absurdities. If an author writes a work at the present time, when authorities have lost almost entirely their weight, it would seem desirable that he confine himself mainly to writing his own opinions and not quoting too largely the opinions of others.

There may be those who will regard this as an additional attraction, as greatly adding to the value of the work; in our opinion, it adds only to the number of pages.

It is right to give to each author his due, and often very well to quote his language, if his sentiment is fully adopted, but hardly worth while to make others say for us what we scarce venture to say for ourselves. The second volume is daily expected and will be noticed in our next number.

---

Physician's Visiting List. Lindsay & Blakiston, Philadelphia.

We have received pocket memorandum for 1869. Its value is well known to the profession and it requires only mention of publication. The following is the contents:

Almanac, Table of Signs, Marshall Hall's Ready Method of Asphyxia, Poisons, and their Antidotes, Table for Calculating the Period of Utero-Gestation. Blank leaves for Visiting List, Monthly Memoranda, Addresses of Patients and others, Addresses of Nurses, their references, etc., Accounts asked for, Memoranda of Wants, Obstetric Engagements, Vaccination Engagements. Record of Births, Record of Deaths, General Memoranda, etc.

---

A Manual on Extracting Teeth: Founded on the anatomy of the parts involved in the operation; the kinds and proper construction of the instruments to be used; the accidents liable to occur from operations, and the proper remedies to retrieve such accidents. By Abraham Robertson, D.D.S., M.D. Second Edition. Philadelphia: Lindsay & Blakiston, 1868.

This work is devoted to a consideration of the diseases of the teeth, the anatomy of the parts concerned, and the manner of extracting them when necessary. It is designed as a guide to the physician in the treatment of the various ills and accidents liable to befall these organs and to prevent him from improprieties in treatment and hasty extraction, when the organs might be preserved by better operative means. It is especially necessary in small towns, where dentists are not located, that the physician to whose care all maladies of the system are submitted, should understand some of the essential facts connected with dentistry. This book will be found eminently suited to the members of the profession, who are consulted sometimes in these matters, and assume, at least, temporarily the care of such cases.

---

Diseases of Children. By Thomas Hillier, M. D. London: Philadelphia, Lindsay and Blakiston, 1868.

This book comprises a series of short monographs, on the more important diseases of children between the ages of two and twelve years. Surgical diseases, and diseases of new-born children, are not included, or of children under two years of age. The following diseases form the topics of the different chapters: Pneumonia, lobar pneumonia, pleurisy, rickets, tuberculosis, diphtheria, acute hydrocephalus, and meningeal tubercle, chronic hydrocephalus, tubercle of the brain and other cerebral affections, pyæmia and otorrhœa, chorea, paralysis, ascetes, scariatina, typhoid fever, skin diseases, epilepsy and convulsions. All these subjects are treated in a masterly manner, and with the view of making the book a complete treatise upon the diseases of children.

As showing the importance of carefully studying the diseases of children, their great frequency, and their enormous mortality, is shown. The author says: "of 1,000 children born, 150 die within twelve months; 113 during the next four years, giving 263, or more than a quarter, within five years of birth. During the next five years 34 die; during the next five years 18 more die; so that at fifteen years of age, only 684 remain of the 1,000 born."

The book is full of clinical observation, and is, throughout, illustrated and enforced by detail of clinical cases. It is a work eminently adapted to practical instruction, and cannot fail to interest all who have the care of children.

Of course, the diseases of children often appear also in adults, but the differences in nature and treatment, render a work exclusively devoted to these diseases, as thus appearing, highly entertaining and instructive.

---

Recherches Expérimentales sur une Nouvelle Fonction du Foie consistant dans la Séparation de la Cholestérine du Sang et Son Élimination sous forme de Stercorine (Séroline de Boudet,) par Austin Flint, fils, Docteur en Médicine.

By a number of interesting and ingeniously conducted experiments the author arrives at the following conclusions, which we translate from the French:

1. Cholesterine exists in the bile, the blood, the nervous substance, the crystalline, and the meconium, but is *not* found in the normal fæces. In blood taken from the arm, we find that the quantity of cholesterine contained in it, is from five to eight times greater than has heretofore been calculated.

2. Cholesterine is formed principally, if not entirely, in the nervous substance, where it is very abundant, and from whence it is carried by the blood and constitutes one of the most important refuse products of the system. Its production is constant, for it always exists in the blood and the nervous substance.

3. Cholesterine is separated from the blood by the liver, thus showing that it is a constant element of the bile, and is diverted into the alimentary canal. The physiology of this substance, both in the blood and in the bile, causes it to be classed with those products which ought to be expelled from the system, or with the number of excretions It preëxists in the blocd—plays no important part in the economy of nature—is expelled by the liver and not manufactured by it—and if its elimination, is disturbed, it accumulates in the system and causes a poisoning of the blood.

4. The bile has two distinct functions which depend on the presence of two elements of totally different characters. One of its functions relates to nutrition. It is due to the presence of the glyco cholate and of the tauro-cholate of soda. These do not preëxist in the blood, but play an important part in the system—and are not expelled from it--are produced by the liver and pertain exclusively to the bile—do not accumulate in the blood when the functions of the liver are deranged, and constitute, in a word, the products of *secretion.* But the liver has another function of a purifying nature, due to the presence of cholesterine, which is an *excretion.* The flowing of the bile is remittent; it is considerably augmented during digestion, but takes place also during the intervals, in order to separate the cholesterine from the blood which it constantly receives.

5. The normal fæces contain, *not* cholesterine, but *stercorine,* (formerly called seroline, because it was supposed to exist only in serum of the blood,) produced by a transformation of the cholesterine of the bile during the act of digestion.

6. The transformation of cholesterine into stercorine does not take place when digestion is suspended, or before it has been established; consequently we find stercorine neither in the meconium nor in the fæces of hibernating animals during their torpid state. These matters contain cholesterine in great abundance, and it is also found in the fæces of animals after a fast of long duration. It is under the form of stercorine that cholesterine is evacuated.

7. The difference between the two varieties of jaundice with which we are familiar, is this: the one is simply characterized by a yellow color of the skin, and is comparatively inoffensive. The other is accompanied by acute symptoms—almost always proves fatal, and depends in one case upon some obstacle to the free flowing of the bile, and in the other to its *total* suppression. In the first case, the bile is retained in the excretory ducts, and its colorlng matter absorbed, whilst in the second, the cholesterine is retained in the blood, and there acts as a poison.

8. There exists a pathological state of the blood, which depends upon the accumulation of cholesterine, and we have named it cholesterimy. It is only produced in the case of an organic change having taken place in the liver, which prevents it from accomplishing its functions as an excretive organ. It is characterized by grave symptoms, which can be traced to the brain, and which depend npon the poisonous effects upon that organ of the accumulated cholesterine. It may or may not be accompanied by jaundice.

9. Cholesterimy does not take place in every case of disease affecting the structure of the liver. To produce it, it is necessary that the disease be of long enough duration to prevent a sufficient elimination of cholesterine. In a case where the disorder be but slight, the healthy part of the organ can fulfill the eliminating function of the whole.

10. In a case of simple jaundice, where the fæces are discolored, and where the bile has no access to the intestine, we find no stercorine in the bile. But in a case of jaundice with cholesterine one can meet with cholesterine, (though always in much reduced proportions,) which denotes an insufficient elimination of the cholesterine of the blood; however, its excretion is not entirely suspended.

